# Long‐term impact of overnight shiftwork implementation on pediatric residents' mental wellness: A repeated cross‐sectional survey

**DOI:** 10.1002/1348-9585.12349

**Published:** 2022-07-29

**Authors:** Hiro Nakao, Osamu Nomura, Mitsuru Kubota, Akira Ishiguro

**Affiliations:** ^1^ Department of General Pediatrics and Interdisciplinary Medicine National Center for Child Health and Development Setagaya‐ku Japan; ^2^ Center for Postgraduate Education and Training National Center for Child Health and Development Setagaya‐ku Japan; ^3^ Department of Emergency and Disaster Medicine Hirosaki University Hirosaki Japan

**Keywords:** Center for Epidemiologic Studies Depression scale (CES‐D), Maslach burnout inventory (MBI), pediatric resident, shiftwork, wellness, work style

## Abstract

**Objectives:**

The Japanese government partially enacted the “Work Style Reform Bill” in 2019. The National Center for Child Health and Development (NCCHD) introduced an Overnight Call Shift (OCS) system for pediatrician training. We conducted a follow‐up survey in 2019 to investigate the long‐term effectiveness of the OCS system to improve the pediatric residents' mental wellness at NCCHD.

**Methods:**

We conducted a questionnaire‐based cross‐sectional survey for pediatric residents in 2019 to compare the data with those of the previous survey in 2012. The questionnaire includes demographic data, working conditions data, and mental wellness assessment by the Center for Epidemiologic Studies Depression scale (CES‐D) and the Maslach Burnout Inventory (MBI).

**Results:**

The collection rate for the 2019 survey was 94.5% (37 participants/39 eligible). Compared to 2012, there were no significant changes in demographic data and working hours, a significant increase by about 30% in residents who took daytime off *after* night work, about 10% decrease in residents who scored 16 and above on the CES‐D, and a significant decrease in the mean score for depersonalization (DP) in the MBI. Multiple regression analyses showed that daytime off *after* night work was the decreasing factor for CES‐D and Emotional exhaustion (EE).

**Conclusions:**

The overnight shiftwork system shortened the pediatric residents' duty hours somewhat, and imposed an impact on the pediatric residents' mental wellness.

## 1 INTRODUCTION

Initiatives to improve wellness and prevent pediatric resident burnout have been attracting attention worldwide to maintain the quality of medical care and secure patient safety.^1^, ^2^, ^3^, ^4^, ^5^ Fatigue and sleep deprivation due to shiftwork adversely affect the alertness and performance of the trainee physicians. Whereas the study groups in the United States have investigated the impact of optimizing the work shift on the residents' fatigue and sleep deprivation, and patient outcomes,^6^, ^7^, ^8^, ^9^, ^10^ there are only a few reports from Asian countries.^11^, ^12^

The single institution survey conducted at the National Center for Child Health and Development (NCCHD) in Japan showed that the working hours of the pediatric residents exceeded 75 hours per week in 2010.^11^, ^12^ In 2019, the Japanese government partially enacted the “Work Style Reform Bill”, and the overtime work hour regulations for physicians are scheduled to be enforced in 2024. The Ministry of Health, Labour, and Welfare has stated that the goal of “work style reform” is to “create a society that allows people to choose a preferred work style that suits their circumstances”.^13^ With the increase in the number of pediatricians with diverse backgrounds in Japan,^14^, ^15^ it is necessary for the faculties of the postgraduate training programs to prepare flexible educational environments that allow for the diverse work styles of the residents.^16^

NCCHD implemented an Overnight Call shift (OCS) system to improve the working environment and wellness (i.e., mental health) of the pediatric residents in 2011. As shown in Table 1, the OCS system allows night‐work physicians to take daytime off, which was not present in the traditional shift system. Although the residents' working hours decreased 1 year after the OCS implementation, the frequency of depressive symptoms did not change during the short‐term duration (i.e., a year).^11^, ^12^ In the current study, we conducted a follow‐up survey at NCCHD in 2019 to investigate the long‐term effectiveness of the OCS system to improve the pediatric residents' mental wellness, including the frequency of depressive symptoms and burnout.

**TABLE 1:** Shift time work schedule

	Time	8	9	16	17	18	7	8	9
Traditional shift system	Work	MC	Daytime work		HO	Night work		MC	Off
Overnight Call Shift (OCS) system		MC	Off		HO	Night work		MC	Off

*Note*: The previous traditional shift system and the Overnight Call Shift (OCS) system implemented in 2011 at NCCHD are shown. In the traditional shift system, physicians who have worked during the daytime work directly at night. In the OCS system, physicians who have taken daytime off work at night.

Abbreviations: HO, hand‐off meeting; MC, morning conference.

## 2 MATERIALS AND METHODS

### 2.1 Study site and participants

NCCHD is a 490‐bed hospital specializing in children and perinatal care. It offers a three‐year clinical training program for pediatric residents to become pediatric specialists in accordance with the regulations of the Japan Pediatric Society, with 10–14 residents per each resident year. They work night shifts in the general pediatric ward rotation, which is the core content of their training.

### 2.2 Design

We distributed a questionnaire‐based cross‐sectional survey to 39 pediatric trainees enrolled in 2019 and compared the data to a prior 2012 study of 42 pediatric residents.^11^ We used the Survey Monkey® online platform for the data collection. We obtained the data online and anonymously, removed responses with missing data, and checked one by one for unnatural data to reduce reporting bias and eliminate malicious responses. To avoid the identification of individuals from the answers in a small group survey, we have taken care to ensure that the researchers who did not directly interact with the residents themselves were put in charge of checking and summarizing the anonymized raw data into statistical values.

### 2.3 Data collection

We gathered the following demographic data: age, sex, marital status, resident year, and postgraduate year. Working hours per week, number of night or holiday duty shiftwork periods per month, number of days off per month, and compliance with the OCS system were also asked to determine their working conditions. To assess mental wellness, we used the Center of Epidemiologic Studies Depression scale (CES‐D)^17^ and Maslach Burnout Inventory (MBI),^18^ which are widely used for screening depression and burnout, respectively. The cutoff of CES‐D is 16 points, and a score of 16 and above is considered to indicate depressive symptoms. There are three components of MBI: Emotional Exhaustion (EE), Depersonalization (DP), and Personal Accomplishment (PA).^19^

### 2.4 Analysis

Statistical analysis methods included Student's *t*‐test for continuous variables and Fisher's exact test for categorical variables, with *p* < .05 considered as statistically significant. We also conducted multiple regression analyses with CES‐D and subscales of MBI as outcome variables for all participants. We used EZR (version 1.54) statistical software,^20^ which is based on R and R commander, for the analysis.

### 2.5 Ethical statement

The study data was anonymized and approved by the Ethics Committee of NCCHD (No. 2318). Online consent was obtained from all survey participants.

## 3 RESULTS

The response rate for this 2019 survey was 94.5% (37 participants/39 eligible). We compared their data with the data from the previous 2012 survey (response rate 97.6%; 41/42).^11^

### 3.1 Demographics and working conditions data

There were no differences in the demographics, including age, gender, marital status, and postgraduate training years, and working conditions data, such as work time per week and numbers of night shifts per month, between 2012 and 2019 (Table 2). Table 3 shows the residents' compliance with the OCS system. Although there was no change in the percentage of the residents responding that they could take daytime off before night work (70.7% vs. 70.3%, *p* = 1.00) the percentage of the residents who could take daytime off after night work increased significantly in 2019 (31.7% vs. 64.9%, *p* < .01).

**TABLE 2:** Demographic and working conditions data

	2012 (*n* = 41)	2019 (*n* = 37)	*p*
Age, years, mean (*SD*)	29.0 (1.9)	29.5 (2.4)	.31
Male, % (*n*)	56.0 (23)	45.9 (17)	.50
Married, % (*n*)	31.0 (13)	29.7 (11)	1.00
1st year resident, % (*n*)	26.8 (11)	32.4 (12)	.63
2nd year resident, % (*n*)	36.6 (15)	27.0 (10)	.47
3rd year resident, % (*n*)	36.6 (15)	29.7 (11)	.63
Postgraduate training, years, mean (*SD*)	4.0 (0.9)	4.4 (1.3)	.75
Work time, hours/week, mean (*SD*)	64.9 (8.8)	66.7 (8.2)	.36
Night or holiday duty shiftwork, periods/month, mean (*SD*)	3.9 (1.6)^a^	4.8 (0.9)^b^	‐
Off‐duty days, days/month, mean (*SD*)	3.8 (1.4)	4.1 (1.1)	.34

*Note*: *p* values were obtained by t‐test for age, postgraduate training, work time, and off‐duty days and Fisher's exact test for male, married and 1st, 2nd, and 3rd year resident.

Abbreviation: *SD*, standard deviation.

^a^ Night shift only.

^b^ Night shift & holiday duty work.

**TABLE 3:** Compliance with the Overnight Call Shift system, % (*n*)

		2012 (*n* = 41)	2019 (*n* = 37)	*p*
Residents who are able to take:	Daytime off *before* night work	70.7% (29)	70.3% (26)	1.00
	Daytime off *after* night work	31.7% (13)	64.9% (24)	<.01

*Note*: *p*‐values were obtained by Fisher's exact test.

### 3.2 Mental wellness

Table 4 shows the CES‐D and MBI data in 2012 and 2019. Although no statistical significance was found in the frequency of the residents showing depressive symptoms (i.e., CES ≥16) and CES‐D score, both frequency (31.7% vs. 20.5%, *p* = .31) and mean score (12.4 vs. 10.3, *p* = .17) were slightly improved in 2019. Furthermore, in the MBI, the mean DP subscale score significantly decreased in 2019, compared to the score in 2012 (9.4 vs. 10.8, *p* = .04). The mean EE (13.5 vs. 12.0, *p* = .13) and PA (17.2 vs. 17.0, *p* = .78) subscale scores were similar between the two groups. Multiple regression analyses showed that daytime off *after* night work was the decreasing factor for CES‐D and EE, and marriage was also the decreasing factor for CES‐D (Table 5).

**TABLE 4:** Center for epidemiologic studies depression scale and maslach burnout inventory

	2012 (*n* = 41)	2019 (*n* = 39)	*p*
CES‐D			
Residents with CES‐D ≥ 16, % (*n*)	31.7 (13)	20.5 (8)	.31
CES‐D score, mean (*SD*)	12.4 (7.5)	10.3 (5.8)	.17
MBI			
Emotional exhaustion, mean (*SD*)	13.5 (4.0)	12.0 (3.2)	.13
Depersonalization, mean (*SD*)	10.8 (3.7)	9.4 (2.2)	.04
Personal accomplishment, mean (*SD*)	17.2 (4.2)	17.0 (4.2)	.78

*Note*: *p* values were obtained by *t*‐test for CES‐D score and each MBI score and Fisher's exact test for residents with CES‐D ≥ 16.

Abbreviations: CES‐D, Center for Epidemiologic Studies Depression scale; MBI, Maslach Burnout Inventory; *SD*, standard deviation.

**TABLE 5:** Multiple regression analyses with CES‐D and subscales of MBI (EE, DP, and PA) as outcome variables for all participants

Outcome variable	CES‐D		Emotional exhaustion	
Predictive variable	β (95% CI)	*p*	β (95% CI)	*p*
Investigation year	−1.31 (−4.49 ~ 1.88)	.42	−0.88 (−2.6 ~ 0.84)	.31
Age	−0.19 (−1.00 ~ 0.61)	.63	0.04 (−0.4 ~ 0.47)	.87
Sex	−0.58 (−3.73 ~ 2.57)	.72	−1.34 (−3.04 ~ 0.37)	.12
Married	−3.76 (−7.14 ~ −0.37)	.03	0.01 (−1.82 ~ 1.85)	.99
Resident year	−1.25 (−4.42 ~ 1.92)	.43	−0.65 (−2.36 ~ 1.07)	.46
Post graduate training	1.98 (−0.76 ~ 4.72)	.15	1.47 (−0.01 ~ 2.96)	0.051
Work time	0.11 (−0.06 ~ 0.29)	.20	−0.02 (−0.11 ~ 0.07)	.67
Off‐duty days	0.12 (−1.13 ~ 1.36)	.85	−0.13 (−0.81 ~ 0.54)	.69
Daytime off *before* night work	−1.09 (−4.81 ~ 2.63)	.56	0.08 (−1.93 ~ 2.1)	.93
Daytime off *after* nightwork	−3.74 (−7.22 ~ −0.26)	.036	−1.96 (−3.85 ~ −0.08)	.041
Outcome variable	Depersonalization		Personal accomplishment	
Predictive variable	β (95% CI)	*p*	β (95% CI)	*p*
Investigation year	−1.37 (−2.93 ~ 0.20)	.085	−0.13 (−2.31 ~ 2.06)	.91
Age	0.01 (−0.39 ~ 0.41)	.96	0.01 (−0.55 ~ 0.56)	.98
Sex	−0.44 (−1.99 ~ 1.11)	.57	−0.48 (−2.65 ~ 1.69)	.66
Married	−0.66 (−2.33 ~ 1.00)	.43	0.47 (−1.86 ~ 2.79)	.99
Resident year	−0.09 (−1.65 ~ 1.46)	0.90	−0.65 (−2.83 ~ 1.53)	.55
Post graduate training	0.62 (−0.73 ~ 1.97)	.36	0.35 (−1.54 ~ 2.23)	.72
Work time	0.04 (−0.05 ~ 0.13)	.35	0.02 (−0.10 ~ 0.14)	.79
Off‐duty days	−0.25 (−0.86 ~ 0.37)	.42	−0.55 (−1.41 ~ 0.31)	.20
Daytime off *before* night work	0.35 (−1.48 ~ 2.18)	.70	−0.42 (−2.97 ~ 2.14)	.75
Daytime off *after* nightwork	−0.86 (−2.57 ~ 0.85)	.32	0.01 (−2.38 ~ 2.41)	.99

*Note*: *p* values were obtained by *t*‐test.

Abbreviations: CES‐D, Center for Epidemiologic Studies Depression scale; MBI, Maslach Burnout Inventory; β, partial regression coefficient; 95%CI, 95% confidence interval.

## 4 DISCUSSION

This study investigated the long‐term impact of implementing the OCS system at our institution as the pediatric residents' mental wellness improvement initiative. We found that one element (i.e., DP) of the burnout scale decreased, and the residents' compliance with the OCS system improved significantly 8 years after the initiative implementation. Multiple regression analyses demonstrated daytime off after night work as the decreasing factor for CES‐D and EE, one subscale of MBI.

In our previous study focusing on the short‐term outcomes of the initiative in 2011, we confirmed that the OCS system enabled us to reduce the residents' work hours from 75 hours to 65 hours per week by restricting them from working before the shift on the day of the night duty. However, residents could not take daytime off after night work due to the high workload related to workforce shortages, and there was no improvement in the frequency of depressive symptoms in the residents (32.3% vs. 31.7%).^11^, ^12^ The Japanese government enacted the “Work Style Reform Bill” in 2019, and this governmental policy could have increased the awareness for the wellness improvement of physicians and driven the establishment of the OCS system in our hospital. Consequently, in 2019, the established OCS system allowed the residents to take daytime off after night work and improve their mental wellness, which was indicated by the improved frequency of depressive symptoms and burnout subscale scores.

In this study, only the DP score in MBI in 2019 decreased significantly compared to that of 2012. DP means the dehumanizing response to the object of emotional labor as a defensive reaction due to mental impoverishment.^19^ EE and PA, the other MBI items besides DP, also showed a tendency to improve without statistical significance. The lack of significant differences may be due to the small number of subjects. In addition, multiple regression analyses suggested daytime off after night work as a decreasing factor for EE and CES‐D. Taken together these results with the improved frequency of depressive symptoms after OCS establishment, we think shiftwork is vital for the residents' mental wellness.

In the United States, the national policy on residents' physician work hours has shifted in recent years. Although the Accreditation Council for Graduate Medical Education (ACGME) prohibited the work shifts exceeding 16 consecutive hours for first‐year residents in 2011, the ACGME reversed its policy allowing shifts of 24 to 28 consecutive hours for all resident physicians in 2017.^8^, ^9^, ^10^ More recently, multi‐centered trials have focused on designing flexible shiftwork to improve residents' wellness and patients' safety.^21^, ^22^ According to educational theories, arranging the learning environment is also important to maintain the well‐being of the trainees.^23^, ^24^ Several studies have demonstrated the relationship between the pediatric residents' learning environment and their frequency of burnout.^1^, ^2^, ^6^ Specifically, the peer mentorship program has been suggested to provide the residents with “safe” training circumstances.^25^, ^26^ Therefore, it may be helpful to implement the peer mentoring program for arranging the residents' learning environment.

There are several limitations to this study. First, this is a single‐center study with a small sample size, and the results cannot be directly generalized to other programs in Japan. However, we believe that it is valuable to share our experiences of the OCS system implementation with other institutions to further improve pediatric residents' well‐being in Japan. In this vein, this study could be a pilot trial for future multi‐institution studies involving pediatric residents in Japan. Second, the repeated cross‐sectional design might have included some confounding factors that affect the study outcomes. In addition, the subjects of each year (2012 and 2019) were not the same residents. However, we have confirmed that there were no significant differences in demographic data between the two groups and conducted multiple regression analyses to remove confounding influences as much as possible to confirm the impact of OCS. Furthermore, the selection process for entering residency and the training program structure have been unchanged during the study period. We therefore consider that the effect of the possible confounding factors on our study results was minimal. Third, in addition to OCS, there may be other changes between the 2012 and 2019 period that affect residents' mental wellness, such as the changes in high workload, interaction with parents or consultants, or supervision of residents, as reported in our previous report.^11^ These changes are difficult to quantify in fact, but we must continue efforts to also survey and improve these factors. Fourth, work style data such as working hours, number of night or holiday duty shiftwork, and number of days off were all self‐reported, because there are no objective records. We have tried to make the questionnaire obtain as concise and accurate data as possible, but it may not be completely accurate. Finally, the measured outcomes in this study were narrowly focused on the depression and burnout scales, whereas the previous studies examined more impactful outcomes, such as physicians' fatigue, sleep disturbance, and patient outcomes.^7^, ^8^, ^9^, ^10^ A multi‐center survey with larger sample size is needed to overcome these limitations and create robust, generalizable evidence.

## 5 CONCLUSIONS

The overnight shiftwork system shortened the pediatric residents' duty hours somewhat, and imposed an impact on the pediatric residents' mental wellness.

The results of this survey were presented at the 124th Annual Meeting of the Japan Pediatric Society in Kyoto, 2021.

## AUTHOR CONTRIBUTIONS

H.N. and O.N. designed and conducted the study, collected and analyzed data, and wrote the manuscript; M.K. and A.I. analyzed data and gave technical support and conceptual advice and edited the manuscript; All authors read and approved the final manuscript.

## DISCLOSURE

The authors declare no Conflict of Interests for this article. This research received no specific grant from any funding agency in the public, commercial, or not‐for‐profit sectors.

## Data Availability

The data that support the findings of this study are available from the corresponding author upon reasonable request.
